# Dysregulation of the Renin-Angiotensin-Aldosterone System (RAA) in Patients Infected with SARS-CoV-2-Possible Clinical Consequences

**DOI:** 10.3390/ijms22094503

**Published:** 2021-04-26

**Authors:** Agnieszka Dettlaff-Pokora, Julian Swierczynski

**Affiliations:** 1 Department of Biochemistry, Faculty of Medicine, Medical University of Gdańsk, 80-211 Gdańsk, Poland; 2 State School of Higher Vocational Education, 75-582 Koszalin, Poland; juls@gumed.edu.pl

**Keywords:** renin-angiotensin-aldosterone system, angiotensin, Angiotensin-Converting Enzyme, SARS-CoV-2

## Abstract

SARS-CoV-2 impairs the renin-angiotensin-aledosterone system via binding ACE2 enzyme. ACE2 plays a key role in the biosynthesis of angiotensin (1-7), catalyzing the conversion of angiotensin 2 into angiotensin (1-7) and the reaction of angiotensin synthesis (1-9), from which angiotensin is (1-7) produced under the influence of ACE (Angiotensin-Converting Enzyme). Angiotensin 2 is a potent vasoconstrictor and atherogenic molecule converted by ACE2 to reducing inflammation and vasodilating in action angiotensin (1-7). Angiotensin (1-9), that is a product of angiotensin 1 metabolism and precursor of angiotensin (1-7), also exerts cell protective properties. Balance between angiotensin 2 and angiotensin (1-7) regulates blood pressure and ACE2 plays a critical role in this balance. ACE2, unlike ACE, is not inhibited by ACE inhibitors at the doses used in humans during the treatment of arterial hypertension. Membrane ACE2 is one of the receptors that allows SARS-CoV-2 to enter the host cells. ACE2 after SARS-CoV-2 binding is internalized and degraded. Hence ACE2 activity on the cell surface is reduced leading to increase the concentration of angiotensin 2 and decrease the concentration of angiotensin (1-7). Disturbed angiotensins metabolism, changes in ratio between angiotensins with distinct biological activities leading to domination of atherogenic angiotensin 2 can increase the damage to the lungs.

## 1. Introduction

SARS-CoV-2 impairs the renin-angiotensin-aledosterone system via binding ACE2 enzyme. ACE2 (Angiotensin-Converting Enzyme 2) is an enzyme that exists in two forms. The dominant form is the long form of the enzyme, which is found on the cell membranes of cells in many organs. Low levels of cell free ACE2 activity was also found in the blood, urine and human cerebrospinal fluid. This form of the enzyme is called the short or soluble form. It is formed from the long outer membrane in a process catalyzed by enzymes from the group of metalloproteinases. ACE2 plays a key role in the biosynthesis of angiotensin (1-7), catalyzing the conversion of angiotensin 2 into angiotensin (1-7) and the reaction of angiotensin synthesis (1-9), from which angiotensin (1-7) is produced under the influence of ACE (Angiotensin-Converting Enzyme). ACE2, unlike ACE, is not inhibited by ACE inhibitors at the doses used in humans during the treatment of arterial hypertension. Membrane ACE2 is one of the receptors that allows SARS-CoV-2 (Severe Acute Respiratory Syndrome Coronavirus 2) to enter the host cells, and consequently the multiplication of the virus and the progression of COVID-19. Binding of SARS-CoV-2 to membrane ACE2 causes internalization and degradation of the enzyme and, consequently, reduction of its activity. This may increase the concentration of angiotensin 2 and decrease the concentration of angiotensin (1-7). This, in turn, can increase the damage to the lungs (and other organs). The soluble form of ACE2 present in the blood also binds SARS-CoV-2. Theoretically, this could prevent the virus from entering the cells and thus prevent it from multiplying. The presence of ACE2 on the cell membranes of cells in organs suggests that these organs may potentially be vulnerable to damage during SARS-CoV-2 infection. Few, sometimes controversial, results from experimental animals and clinical observations suggest that ACE inhibitors and AT1R blockers [1,2,3,4] may induce ACE2 in some organs and, consequently, facilitate virus entry into cells and its multiplication [5,6,7,8]. On this basis, it has been hypothesized that the use of ACE inhibitors and AT1R blockers in the treatment of arterial hypertension during the COVID-19 pandemic may make the patient more susceptible to SARS-CoV-2 infection, and the course of COVID-19 may be more severe. To date, however, there is no scientific evidence that ACE inhibitors or AT1R blockers increase the risk of infection and adversely affect the course of the disease. In conclusion, the research results available so far indicate that SARS-CoV-2 infection is one of the factors that may lead to dysregulation of the RAA system. The clinical consequences of this process require further clinical observations and experimental studies.

The classical system of renin-angiotensin-aldosterone (RAA) is widely recognized as the main regulator of blood pressure and the water and electrolyte balance of the human body. Renin plays an essential role in the activation of this system. Renin, also called angiotensingenase (EC 3.4.23.15), is synthesized as preprorenin which is transformed post-translational into prorenin. This, in turn, is converted to biologically active renin and is secreted by the glomerular cells of the glomerular apparatus. The process of synthesis and secretion of renin is very precisely regulated [9]. Renin catalyzes the hydrolytic detachment of the N-terminus of angiotensinogen (a protein formed mainly in the liver, composed of about 400 amino acids), a decapeptide called angiotensin 1. Then, after the hydrolytic detachment of two amino acids (-histidine-leucine-COOH) from the C-terminus of angiotensin 1, angiotensin 2 is formed-an oligopeptide composed of 8 amino acids (octapeptide), with a strong pressor effect. The process of angiotensin 2 synthesis is catalyzed by the Angiotensin-Converting Enzyme (ACE; EC 3.4.15.1). This enzyme is found in the cell membranes of the vascular endothelium, renal tubular cells (mainly the proximal tubules) and neuroepithelial cells [9].

ACE also catalyzes reactions in which the substrates are other oligopeptides involved directly or indirectly in the regulation of blood pressure, such as bradykinin and kallidin. Bradykinin is a nine amino acid oligopeptide that dilates blood vessels, including the coronary vessels. Kallidin is an oligopeptide of ten amino acids that is proteolyzed into bradykinin and lysine. The effect of ACE on bradykinin is its inactivation, e.g., loss of the ability to dilate blood vessels [10,11]. It follows that the higher the ACE activity, the greater the angiotensin 2 level leading to decreased bradykinin and vessels don’t dilate.

Substrates for ACE are also other oligopeptides, such as angiotensin (1-9) or angiotensin (1-7), oligopeptides related directly or indirectly to the regulation of blood pressure and other important physiological processes.

**Figure 1:**
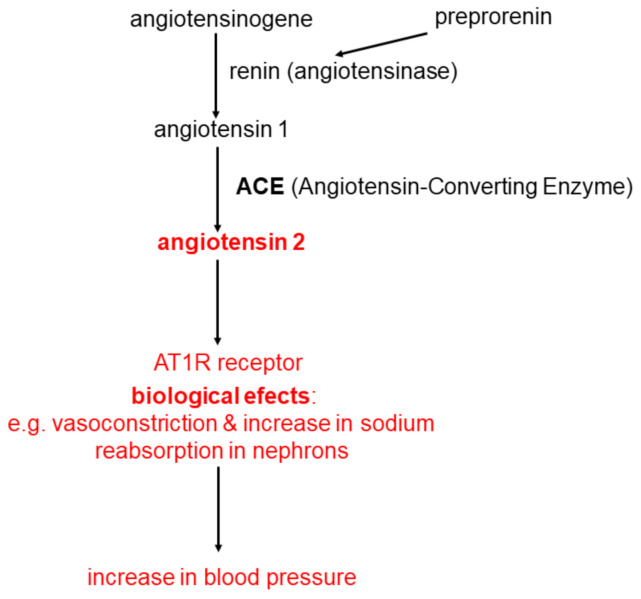
The classical system of renin-angiotensin-aldosterone (RAA).

Angiotensin 2 produced by an ACE-catalyzed reaction binds to the angiotensin 2 receptor (AT1R), which is present mainly in the cell membranes of cells of the cardiovascular system, kidneys, adrenal cortex and the sympathetic nervous system. Binding of angiotensin 2 to AT1R present in the cell membranes of the cardiovascular system causes vasoconstriction. Binding to the AT1R receptor present in renal tubular cells causes an increase in sodium reabsorption in nephrons, which also leads to an increase in blood pressure and blood volume. The binding of angiotensin 2 with AT1R present in the cells of the glomerular layer of the adrenal cortex leads to the stimulation of the activity of aldosterone synthase, which catalyzes the synthesis of aldosterone from deoxycorticosterone according to a simplified scheme: deoxycorticosterone → corticosterone → 18-hydroxycorticosterone → aldosterone. Aldosterone is a major water-electrolyte balance controlling hormone controlling sodium reabsorption in distal nephron. The processes discussed above are presented in Figure 1.

## 2. Angiotensin Metabolism

In the last dozen or so years, the classic RAA system has been expanded with new elements, of which the most important from a clinical point of view seems to be the Angiotensin-Converting Enzyme 2 (ACE2). ACE2 owes its name to the high degree of structural homology with ACE. The structure of ACE2 is approximately 40% identical and approximately 60% similar to ACE [12]. ACE2 has an intrinsic enzymatic function and the function of a receptor that binds SARS-CoV and SARS-CoV-2. It occurs mainly on the cell membrane of cells in organs, such as: heart, blood vessel endothelial cells, renal tubular cells, intestine, testes, oral and nasal mucosa, cornea, oesophagus, stomach, liver, fat tissue and lungs (type 2 pneumocytes and macrophages), however, the expression level of ACE2 varies widely [13,14,15,16]. This suggests that many organs have the potential to be potentially damaged during SARS-CoV-2 infection. This is in line with clinical observations that multiple organs may be damaged in patients infected with the SARS-CoV-2 virus.

ACE2 is a carboxypeptidase that catalyzes the hydrolysis of a wide variety of oligopeptides. It seems that in the human body the most important reactions catalyzed by ACE2 [17,18] are the transformation of:

1. angiotensin 2 to angiotensin (1-7) according to the Equation:
  angiotensin 2 + H_2_O → angiotensin (1-7) + phenylalanine
2. angiotenisin 1 to angiotensin (1-9) according to the Equation:
  angiotensin 1 + H_2_O → angiotensin (1-9) + leucine

Formed in the reaction angiotensin (1-9), whose biological function has not yet been elucidated, may be further converted to angiotensin (1-7) by ACE. From the above data it appears that ACE 2 plays an important role in the synthesis of angiotensin (1-7) catalyzing: (a) mainly the conversion of angiotensin 2 to angiotensin (1-7) and (b) the conversion of angiotensin 1 to angiotensin (1-9), which is converted into angiotensin (1-7) by ACE.

ACE2, converting angiotensin 2 to angiotensin (1-7), plays an important role in regulation of angiotensin 2 level (causes a decrease) and angiotensin (1-7) (causes an increase). Angiotensin (1-7) is predominantly degraded (inactivated) by ACE reaction:

angiotensin (1-7) + H_2_O → angiotensin (1-5) + histidine-proline

It is evident that ACE and ACE2 play key role in angiotensin level regulation (key role of ACE in angiotensin 2 synthesis and ACE2 in degradation). Thanks to significant parts of ACE2 in angiotensin 2 degradation, ACE2 is called negative regulator od RAA system [19]. 

ACE is blocked by inhibitors used in hypertension treatment. In similar dosages ACE2 is not sensitive to these pharmaceuticals (ACE inhibitors do not inhibit ACE2) [20].

**Figure 2:**
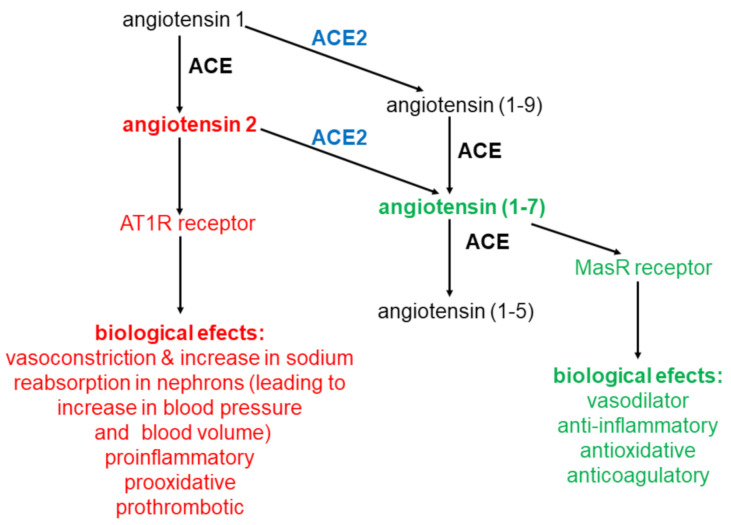
Angiotensins conversions. Metabolism of angiotensins is controlled by a pair of enzymes ACE and ACE2.

Molecular mechanism of angiotensin (1-7) action is connected with Mitochondrial assembly Receptor (MasR). On Figure 2 formation and action of angiotensin (1-7) was shown, together with comparison with formation and action of angiotensin 2. In general angiotensin 2 and angiotensin (1-7) actions are contrary: (a) angiotensin 2 is a vasoconstrictor, while angiotensin (1-7) is a vasodilator; (b) angiotensin 2 is proinflammatory, angiotensin (1-7) anti-inflammatory; (c) angiotensin 2 induces organ fibrosis, angiotensin (1-7) inhibits it; (d) angiotensin 2 is prooxidative, angiotensin (1-7) antioxidative; angiotensin 2 stimulates proliferation, angiotensin (1-7) inhibits it; angiotensin 2 is prothrombotic, angiotensin (1-7) anticoagulatory [21,22]. In general angiotensin (1-7) has a beneficial effect (is a protective for organs oligopeptide), while angiotensin 2 is in destructive one, having negative effect on the human body. The physiological state of equilibrium between ACE and ACE2 activities determines the correct level of angiotensin 2 and angiotensin (1-7). This, in turn, determines the proper functions of the human body. Any disturbance in the activity of these enzymes can lead to serious disturbances in the angiotensin 2/angiotensin (1-7) ratio in the human body, with consequent disturbances in the function of many organs.

## 3. SARS-CoV-2

**Figure 3:**
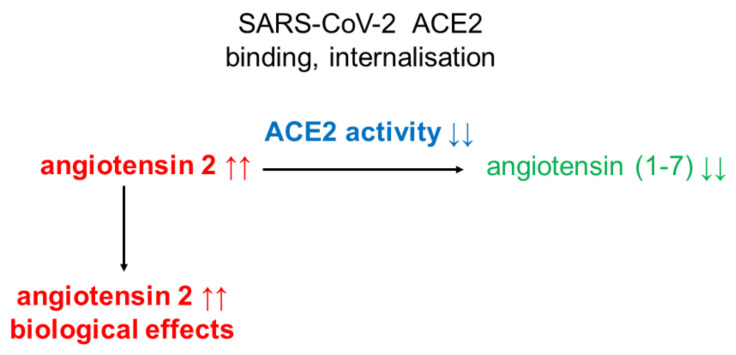
SARS-CoV-2 impacts on angiotensins ratio. SARS-CoV-2 causes inactivation of ACE2 enzyme, leading to negative biological effects of angiotensin 2 only.

One of the factors that may disturb the balance between the levels of angiotensin 2 and angiotensin (1-7), and thus cause dysregulation of the RAA system, is SARS-CoV-2, the virus causing COVID-19. It is an enveloped virus (an envelope made mainly of lipids and proteins), the genome of which is single-stranded RNA (hence the name: RNA virus), of about 30,000 nucleotides. In this respect, it is one of the largest RNA viruses known to date. Colloquially, this virus is called the coronavirus. However, keep in mind that this is one of the family of known coronaviruses. The name comes from the characteristic (solar corona) crown on the surface of the virus. An important component of this crown is a protein that creates protrusions, called spikes. For this reason, this protein is called a spike protein or S protein. It plays a key role in recognizing the receptor on the surface of the cells of a person infected with the virus. As already mentioned, ACE2, present in the cell membranes of cells of many organs, is one of the receptors for this virus entry [23]. In vitro (cell line) and in vivo (animal) studies indicate that ACE2 overexpression facilitates SARS-CoV2 infection and replication [24]. It is worth noting that ACE2 is also a receptor for SARS-CoV, a virus discovered several years ago [25]. At this point, it is important to note a number of similarities between SARS-CoV 2 and SARS-CoV. Noteworthy is the great similarity in the structure of the spike protein (S protein) responsible for the binding of these viruses to ACE2 [26]. However, the affinity of SARS-CoV-2 for ACE2 is many times (10-20 times) higher than that of SARS-CoV [27]. This explains (at least in part) why SARS-CoV-2 is more transmissible than SARS-CoV [23]. Thanks to the Receptor Binding Domain, located in the fusion protein (S protein), SARS-CoV-2 binds to ACE2, which is present on the cell surface of many organs. As a result, the virus fuses with the host cells, and serine protease 2 marked with the symbol TMPRSS2 (transmembrane protease, serine 2), cathepsin B/L or a cell protease called furin (furin) [28] allow the virus to enter the cell and its multiplication (replication) and transmission from cell to cell. At the same time, ACE2 is internalized and degraded inside the infected cell, and consequently its activity is lowered and angiotensin 2 increases (Figure 3). This means that SARS-CoV-2 can significantly impair the function of RAA in patients infected with this virus. By reducing the activity of ACE2, SARS-CoV-2 may cause a significant advantage of angiotensin 2 over angiotensin (1-7), which, in the light of the data presented above, may adversely affect many functions of the human body, for example lung function, causing acute respiratory failure (ARDS). Recent published research results indicate that in SARC-CoV-2 infected patients with developed COVID-19, the blood angiotensin 2 concentration is much higher than in healthy people [29]. Which seems to confirm decreased ACE2 activity. However, it should be noted that these studies were conducted on a relatively small group of patients. Moreover, these studies did not include the activities of angiotensin 2 producing and degrading enzymes (i.e., ACE and ACE2, respectively) [29]. Therefore, there is no clear evidence that decreased ACE2 activity or increased ACE activity is responsible for the increase in angiotensin 2 concentration.

Particular attention should be paid to the advantage of ACE activity over ACE2 and the associated excessive production of angiotensin 2 compared to angiotensin (1-7). Studies on SARS-CoV shown that ACE2 overexpression in mice infected with this virus leads to an aggressive disease course [30]. This condition can lead to ARDS-acute respiratory failure, a condition seen in patients with COVID-19. This is also suggested by the results of experiments in an animal model (mouse) of acute lung injury, where angiotensin 2 plays a key role in this process [31]. This damage is alleviated by blocking the RAA system. The results of these experiments suggest that ACE2 plays an important role not only in SARS-CoV entry into the cell and disease development, but also in protecting the lungs from damage caused by excessive amounts of angiotensin 2. By analyzing these facts only, two contradictory conclusions can be drawn: ACE2 expression may be disadvantageous as it increases the possibility of virus entry into cells (the role of ACE2 as a receptor for SARS-CoV2) and b) an increase in ACE2 expression may be beneficial as it prevents acute respiratory failure by lowering angiotensin 2 and increasing angiotensin (1-7) levels. However, there seems to be no close relationship between the amount (density) of ACE2 on cell membranes and the degree of damage to organs of humans infected with SARS-CoV-2. This is evidenced by the relatively low level of ACE2 expression in the lungs compared to other organs, such as the intestines or kidneys [15], which, according to the data published so far, are the most vulnerable to infection. Probably the lungs are most vulnerable to damage due to the large surface area readily accessible to the virus and the special properties of type II pneumocytes which allow the virus to replicate rapidly [22]. 

Some authors indicate that the plasma ACE2 level is much higher in patients with cardiovascular diseases [32,33,34,35]. Moreover, in animal experiments, ACE2 expression was shown to be significantly elevated in the hearts and kidneys of rats and mice treated with ACE inhibitors or angiotensin 2 receptor blockers [36,37]. Based on these observations, Fang et al. [5] hypothesized that the use of ACE inhibitors or angiotensin 2 receptor blockers in hypertensive patients infected with SARS-COV-2 virus and symptoms characteristic of COVID-19 may be dangerous (even harmful). The authors of this study also suggest that in COVID-19 patients, these drugs should be replaced with calcium channel blockers [5].

The information that ACE inhibitors and AT1R blockers may increase the risk of SARS-CoV-2 infection and adversely affect the course of COVID-19 was disseminated quite quickly by global social media and caused some confusion in the medical community. Probably of great importance in this was the fact that patients with arterial hypertension, heart disease, diabetes and the elderly are the most vulnerable to infection and the severe course of COVID-19 disease [38,39], and at the same time it is a group of patients who, due to arterial hypertension, are very often treated with ACE inhibitors and AT1R blockers.

The basis of Fang and colleagues’ hypothesis is as follows: (a) increase in ACE2 activity in cardiovascular diseases (also in other diseases, e.g., diabetes) and (b) increase in ACE2 activity in experimental models under the influence of ACE inhibitors and angiotensin 2 receptor blockers [5]. Increase in activity, associated with an increase in receptor density on the surface of the host (patient) cells, may facilitate the fusion of the virus with human cell membranes. However, the weakness of this hypothesis is the lack of evidence that ACE inhibitors and angiotensin 2 receptor blockers increase the risk of SARS-COV-2 infection or an unfavorable course of COVID-19 [6]. Moreover, other studies in experimental models and clinical observations do not confirm the increase of ACE2 by ACE inhibitors and angiotensin 2 receptor blockers [7,33] and that despite the increase in ACE2 by ACE inhibitors and receptor blockers in the heart and kidney, there is no increase in ACE in the lungs [36,37]. Another argument against the concept of Fang and colleagues was the result obtained in the acute lung injury model, including the SARS-CoV infection model. These results suggest that angiotensin 2 receptor blockers may attenuate (alleviate) the disease induced by SARS-CoV by blocking the adverse effects of angiotensin 2 by blocking the AT1R (angiotensin 2 receptor) [36]. This receptor (AT1R) plays a key role in angiotensin 2 induction of acute lung injury in the animal-mouse model [31]. The results of these experiments suggest a rather beneficial effect, at least on the lungs, of the use of angiotensin 2 inhibitors and AT1R blockers.

Another argument against the concept of Fang and colleagues are the results of studies that indicate that the level of ACE2 expression is not the highest in the lungs, as it might seem in connection with the COVID-19 pandemic, but in the intestine and kidneys [15]. Presumably, the lungs are the most vulnerable to infection due to the large surface area readily accessible to the virus and the special properties of type II pneumocytes that facilitate SARS-CoV2 multiplication in the lungs [22].

Analyzing the data published so far on the effect of ACE inhibitors or AT1R blockers, two opposite conclusions can be drawn: (a) the increase in the level of ACE2 expression as a result of the use of these drugs may be unfavorable because it increases the possibility of viral entry into cells (the role of ACE2 as a receptor for SARS-CoV2) and (b) increase of the level of ACE2 expression may be beneficial as it prevents acute respiratory failure by lowering the concentration of angiotensin 2 and increasing the concentration of angiotensin (1-7).

Summarizing considerations on the concept of Fang and colleagues, it can be stated that the results of the studies published so far indicate that there is no scientific basis to replace ACE inhibitors or AT1R blockers with other drugs during the treatment of hypertension during the COVID-19 pandemic, because of fear of a higher risk of SARS-CoV-2 infection and a life-threatening course of the disease. It does not mean that this problem is unequivocally resolved and does not require further basic research and clinical observations on the effects of ACE inhibitors and AT1R blockers in patients infected with SARS-CoV2 and COVID-19.

Some proteases, e.g., metalloproteinase 17 (ADAM17-A Disintegrin-like And Metalloprotease 17) are released from cell membranes as a fragment of the enzyme (short form of the enzyme), still showing enzymatic activity, which enters the blood, urine (and other biological fluids like cerebrospinal fluid) [39,40,41,42,43]. Interestingly, the binding of angiotensin 2 to AT1R and its activation increases the activity of ADAM17, which in turn increases the level of the short form of ACE2 in the blood [41]. This form of the enzyme is also called soluble ACE2. Plasma level of this enzyme is very low compared to the activity of the membrane-bound enzyme [8,13,44]. In some pathological conditions, the level of soluble ACE2 (sACE2) increases significantly in the blood, e.g., in arterial hypertension [41]. In urine, the level of sACE2 can be significantly higher than in blood, and the source of the urine enzyme is the proximal tubular membrane [45]. However, it should be remembered that the vast majority of ACE2 activity is associated with cell membranes. This form of the enzyme is immeasurable, it is practically impossible to measure it in vivo in humans, and therefore is unsuitable for diagnostic purposes. In humans, the soluble form of ACE2 (sACE2) is quantifiable and it (as previously mentioned) represents only a few percent of the total enzyme activity. For this reason, it does not represent the total activity of ACE2 and its function in the human body.

Some researchers suggest that the soluble form of ACE2 may prevent the virus from entering cells (it may be a useable trap for SARS-CoV-2), and thus inhibit its multiplication and disease progression. This is due to the binding of the virus in the blood by soluble ACE2 and blocking its entry into the host cell [45]. This idea seems unlikely due to the very low blood levels of ACE2. However, the concept of treating acute respiratory failure (ARDS) in COVID-19 patients with human recombinant ACE2 (rhACE2) seems to be theoretically justified due to viral binding and angiotensin (1-7) synthesis. Especially since human recombinant ACE2 (e.g., APN01) has already been tested on relatively small groups of patients with ARDS. It was shown that administered rhACE2 quickly lowers the concentration of angiotensin 2 [46,47,48]. 

It is worth noting that sACE2 binds SARS-CoV2 similarly to the membrane enzyme [49]. In the context of the development of the COVID-19 pandemic, it is worth to emphasize that the binding of SARS-CoV2 to membrane and to soluble ACE2 has opposite effects. Binding of SARS-CoV2 to the membrane ACE2 allows the virus to enter the cell, multiply and, consequently, cause the disease. In turn, binding of SARS-CoV2 to sACE2 blocks the entry of the virus into the cell (sACE2 is a trap for SARS-CoV2) and consequently, potentially prevents the progression of COVID-19.

By catalyzing the conversion of angiotensin 2 to angiotensin (1-7), ACE2 has a protective effect on the lungs, as it lowers the concentration of angiotensin 2 (a peptide worsening pulmonary function) and increases the concentration of angiotensin (1-7) (a peptide that improves lung function). These two processes: (a) binding the virus (hindering its entry into the cell) and (b) the production of angiotensin (1-7) with the simultaneous degradation of angiotensin 2 (angiotensin 2 → angiotensin (1-7)), became the basis of the concept according to which recombinant human sACE2 (rhACE2) may become a potential drug for severe lung injury (ARDS) in the course of COVID-19. It is worth mentioning here that human recombinant ACE2 (e.g., APN01) have already been studied in relatively small groups of patients with ARDS. Administered rhACE2 rapidly lowered the concentration of angiotensin 2 [46,47,48].

An increase in the expression of the gene encoding ACE2 (measured by protein level) was also found in the heart and kidneys of rabbits with experimentally induced atherosclerosis, treated with statins (atorvastatin) [50]. Treatment with fluvastatin in experimental diabetic rats also causes an increase in ACE2 expression (protein level) in the heart [51]. PPARγ (rosiglitazone) agonists also increase expression of the gene encoding ACE2 (protein level) in the aorta of experimental hypertension rats [52]. On this basis, it can be assumed that some other drugs may have, as a side effect, a stimulating effect on the expression of the ACE2 gene and, consequently, disrupt the RAA system. In theory, these drugs can also affect the course of SARS-CoV-2 infection and the progression of COVID-19.

## 4. Conclusions

The available research results indicate that: (a) ACE2, as one of the SARS-CoV-2 receptors, may play an important role in SARS-CoV-2 infection and progression of COVID-19; (b) SARS-CoV-2 infection may be one of the factors leading to a change in ACE2 activity and, consequently, to disorders of the RAA system. The clinical consequences of RAA disorders in the progression of COVID-19 are not entirely clear and require further clinical observations and experimental studies. Moreover, the published results of studies presented in this review indicate that some commonly used drugs (such as ACE inhibitors, AT1R blockers and statins) may influence ACE2 activity and, consequently, the RAA system. For now, there is no need to replace ACE inhibitors or AT1R blockers with other drugs (during the treatment of arterial hypertension) during the COVID-19 pandemic for fear of a higher risk of SARS-CoV-2 infection and a more life-threatening course of the disease. It does not mean that this problem is unequivocally resolved and not requires further basic research and clinical observations on the effects of ACE inhibitors and AT1R blockers (and other drugs, e.g., statins) in patients infected with SARS-CoV2 and COVID-19.
